# Pigmentation Effect of Rice Bran Extracted Minerals Comprising Soluble Silicic Acids

**DOI:** 10.1155/2016/3137486

**Published:** 2016-11-01

**Authors:** Hyun-Jun Jang, Young-Kwon Seo

**Affiliations:** Department of Medical Biotechnology (BK21 Plus team), Dongguk University, Seoul, Republic of Korea

## Abstract

Our investigation focused on identifying melanogenesis effect of soluble minerals in rice bran ash extract (RBE) which include orthosilicic acid (OSA). Melanocytes were apparently normal in terms of morphology. It was, however, shown that they were stressed a little in the RBE and OSA added media in aspect of LDH activity. Melanin synthesis and intracellular tyrosinase activity were increased by treatment of RBE which is similar to that of OSA. The Western blotting results showed that TRP-1, tyrosinase, and MITF expression levels were 2-3 times higher in the OSA and RBE groups compared to the control group which promoted melanin synthesis through CREB phosphorylation. Moreover, histology and immunohistochemistry were shown to have similar result to that of protein expression. As a result, minerals which comprise orthosilicic acid has the potential to promote melanogenesis and both RBE and OSA have similar cell viability, protein expression, and immunostaining results, suggesting that RBE comprises specific minerals which promote melanin synthesis through increasing of MITF and CREB phosphorylation. Therefore, RBE could be used as a novel therapeutic approach to combat melanin deficiency related diseases by stimulating melanocytes via its soluble Si and mineral components.

## 1. Introduction

Melanin deficiency diseases, such as canities and vitiligo, primarily result from dysfunction of the melanocytes or cell aging; thus many therapies to treat and/or stop the progression of melanin deficiency diseases have been investigated.

Numerous studies have revealed that various natural extracts promote melanocyte activation. Hong et al. reported that black soybean extracts have melanogenic effects in Melan-a cells, which increase melanin contents and intracellular tyrosinase activity approximately 30 and 20%, respectively [1]. Similarly, An et al. investigated the melanogenesis effect of* Cornus officinalis *extract in Melan-a cells.* Cornus officinalis *extract-treated cells had 36.1% more melanin content and upregulated expression of tyrosinase, tyrosinase-related protein 1 (TRP-1) and 2 (TRP-2), and microphthalmia-associated transcription factor (MITF) [2]. Furthermore, Park et al. showed that the extract of* Zingiber cassumunar *Roxb. had a melanogenesis effect in B16F10 melanoma cells. The components enhanced extracellular signal-regulated kinase (ERK) activation, tyrosinase activity, and upstream stimulating factor-1 (USF1) levels. Additionally, melanin synthesis was decreased in USF1-knockdown cells [3].

Similar to these above-mentioned studies, many researchers have used organic materials to stimulate melanogenesis. However, to the best of our knowledge, the use of inorganic mineral components to stimulate melanocytes has not been published. Rice bran ash extract (RBE) has had its organic compounds removed by incineration, leaving organic compounds, such as Si, K, P, and soluble silicic acids.

Silicic acid exists in various forms, including a family of compounds with the formula [SiO_*x*_(OH)_4−2*x*_]_*n*_, such as silicon dioxide (SiO_2_). Most of these compounds are water-insoluble, causing cytotoxicity* in vivo*. Among them, however, orthosilicic acid (OSA,Si(OH)_4_) (OSA) is water-soluble and has low toxicity compared to insoluble silicic acids [4, 5].

The effects of OSA have been investigated in various cell types by numerous researchers. Reffitt et al. reported OSA promoted collagen 1 synthesis and increased the expression of alkaline phosphatase and osteocalcin in osteosarcoma cell line MG-63 [6, 7]. In an aged, ovariectomized rat, OSA supplementation increased femoral and lumbar bone mineral density by decreasing Ca excretion and slowing down bone turnover [8]. Also, the combination of OSA and Ca/vitamin D3 increased bone mineral density compared to Ca/vitamin D3 alone in a double-blind placebo-controlled clinical study [9], suggesting that OSA shows potential as a new treatment for bone-related diseases, such as osteoporosis.

In addition to its benefits in bone-related diseases, Calomme and Vanden Berghe reported that OSA intake increased collagen concentration by approximately 10% in dermis and cartilage [10], suggesting that Si maybe involved in both extracellular matrix formation and Ca metabolism. Also, Grotheer et al. showed that OSA had anti-inflammatory properties in chronic wounds [11] and Barel et al. reported that the positive effects of OSA, on skin, hair, and nails, increased with the increase in hydroxyproline concentration in the dermis [12].

Whilst the use of natural materials to promote bone, skin, and cartilage repair has been extensively studied and the organic compounds and minerals from RBE have been used for cell activation, to the best of our knowledge, no studies have been published on the use of mineral components to stimulate melanocytes or promote melanogenesis. Therefore, this study investigated the pigmentation-promoting effect of OSA and the combination of OSA with RBE, which contains mineral components, such as soluble Si.

## 2. Materials and Methods

### 2.1. Material

RBE was obtained from the carbonized chaff of rice bran. Rice bran ash (200 g) was added to 1 L of distilled water and stirred at 400 rpm and 100°C for 24 h. After that, the mixture was filtered through 10 *μ*m filter paper, centrifuged at 10000 rpm at 25°C for 30 min to remove any remaining particulate matter, and then sterilized using a 0.2 *μ*m syringe filter. OSA was obtained from Natural Factors, BioSil, USA.

### 2.2. Cell Culture

QualiCell human melanoblasts (Creative Bioarray, New York, USA) were cultured in medium 254 (M254, Invitrogen, Waltham, USA) containing PMA-free human melanocyte growth supplement (HMGS-2, Invitrogen, Waltham, USA), 50 units/mL penicillin, and 50 *μ*g/mL streptomycin (HyClone, USA) by incubation in an 80 mm dish at 37°C in a humidified atmosphere with 5% CO_2_.

### 2.3. Melanin Content Determination

Melanoblasts were seeded at 1 × 10^4^ cells/well into 6-well plates and cultured for 3  days in differentiation media containing HMGS-2, 10 nM *α*-melanocyte-stimulating hormone (*α*-MSH, Sigma-Aldrich), 10 nM 12-O-tetradecanoylphorbol-13-acetate (TPA, Sigma-Aldrich), and 20 *μ*M forskolin (Sigma-Aldrich). Then, the media was exchanged for M254 media containing 0.24 *μ*g/mL OSA and 30 *μ*g/mL RBE and the differentiated melanocytes were cultured for further 3 days.

The melanin contents in melanocytes were determined using a previously published method [13] with modifications. After removing the culture media from the plates, the cells were lysed with 10% DMSO solution, dissolved in 1 M NaOH, and boiled at 80°C for 2 h. Then, the cells were centrifuged at 15000 rpm for 15 min. The melanin content of the supernatant was measured using an ELISA plate reader at 405 nm (Spectrum Analyzer, Victor 1420-050, PerkinElmer Life Sciences, Turku, Finland).

### 2.4. Tyrosinase Activity Determination

Intracellular tyrosinase activity was measured using a previously described method [13] with slight modifications. Melanoblasts were seeded at 1 × 10^4^ cells/well in 6-well plates and cultured for 3 days in differentiation media. Then, the medium was exchanged for M254 containing 0.24 *μ*g/mL OSA and 30 *μ*g/mL RBE and the differentiated melanocytes were cultured for further 3 days. After removing the culture media from the plates, the cells were washed with PBS and lysed with 10% triton X-100 (Sigma-Aldrich). The cells were centrifuged at 15000 rpm for 15 min, and then the protein content of the supernatants was measured by the bicinchoninic assay (BCA) (Thermo Fisher Scientific, USA). 3,4,-Dihydroxyphenylalanine (L-DOPA) (Sigma-Aldrich) (10%) was dissolved in sodium phosphate buffer (10 mM). After 30 min incubation at 37°C, absorbance was measured at 475 nm using an ELISA plate reader (PerkinElmer Life Sciences).

### 2.5. Cell Damage Evaluation

Cell damage was evaluated by the lactate dehydrogenase (LDH) assay. Aliquots (100 *μ*L) of the media obtained after culturing the cells for 3 days in M254 containing OSA and RBE (above-mentioned) were placed in a 96-well plate, 50 *μ*L of an LDH-LQ kit solution (Asan Pharmaceutical Inc., Korea) was added, and then the plates were incubated at 25°C for 30 min. The reaction was stopped by adding 50 *μ*L 1N HCl and then absorbance was measured at 490 nm.

### 2.6. Western Blotting

Human-derived melanoblasts were seeded in 60 mm dishes (*N* = 3) at a density of 1 × 10^5^ cells and incubated for 3 days. The medium was removed, and the cells were washed twice with phosphate-buffered saline (PBS) and lysed in PBS containing 10% glycerol, 5%  *β*-mercaptoethanol, 2% sodium dodecyl sulfate (SDS), and 0.01% bromophenol blue in a 62.6 mM Tris-HCl buffer (pH 6.8). The cell lysates were then denatured at 100°C for 5 min. The protein content of the cell lysates was quantified by the BCA assay, and equal amounts of protein per sample were separated by 10% SDS-polyacrylamide and electrotransferred onto a nitrocellulose membrane (Millipore Co., Massachusetts). The membranes were blocked in 5% fat-free skim milk dissolved in Tris-buffered saline (TBS) containing 0.1% Tween 20 (TBS-T buffer) at room temperature for 1 h. After washing with TBS-T, the membrane was incubated for 1 hour in 10% bovine serum albumin, containing primary antibodies: anti-mouse *β*-actin antibody (1 : 2000), anti-goat TRP-1 antibody (1 : 500), anti-goat tyrosinase antibody (1 : 500), anti-rabbit MITF antibody (1 : 500), anti-rabbit CREB (cAMP-responsive element binding protein) antibody (1 : 1000), and anti-rabbit P-CREB (phosphorylated-CREB) antibody (1 : 1000). The primary antibodies were removed; bound primary antibody was detected by applying a horseradish peroxidase- (HRP-) conjugated anti-rabbit, anti-goat, and anti-mouse secondary antibody for 2 h at room temperature. The membrane was washed in TBS-T; the blot was visualized with enhanced chemiluminescence reagent (Thermo Fisher Scientific, USA) and photographed using a gel imaging system, ChemiDoc XRS+ (Bio-Rad, Hercules, CA, USA). The results were quantified using ImageJ software (National Institutes of Health, Bethesda, MD, USA).

### 2.7. Immunohistochemistry

Immunohistochemistry was conducted after culturing the cells for 3 days in M254 media containing OSA and RBE (above-mentioned). Monolayer cultured melanocytes were fixed in 4% buffered formaldehyde solution and then incubated with anti-melanoma monoclonal antibody HMB45 solution (1 : 1000). Localization of HMB45 was performed through an avidin-immunoalkaline phosphatase method with vector red (Vector Laboratories, Burlingame, CA), which is a red chromogen product critical to the success of immunolocalization in pigmented tissues. Microscopic images were captured with a Nikon digital camera attached to a Nikon Optiphot-2 microscope [14].

### 2.8. Histochemical Evaluation

The histochemical evaluation was conducted after culturing the cells for 3 days in M254 media containing OSA and RBE (above-mentioned). The samples were fixed in 4% paraformaldehyde, sectioned, and stained with Fontana-Masson silver nitrate (Kojima Chemical, Kashiwabara, Japan) for 1 h at 56°C and then washed with distilled water and fixed in 5% sodium thiosulfate solution (Duksan, Seoul, Korea) for 5 min and washed with distilled water. After that, samples were stained with nuclear fast red solution (Fluka, Buchs, Switzerland) for 5 min and washed with distilled water 3 times. Finally, the samples were dehydrated with 95% and then 100% ethanol and then washed with xylene (Duksan) 2 times [14].

## 3. Results

### 3.1. Evaluation of Cell Damage

**Figure 1:**
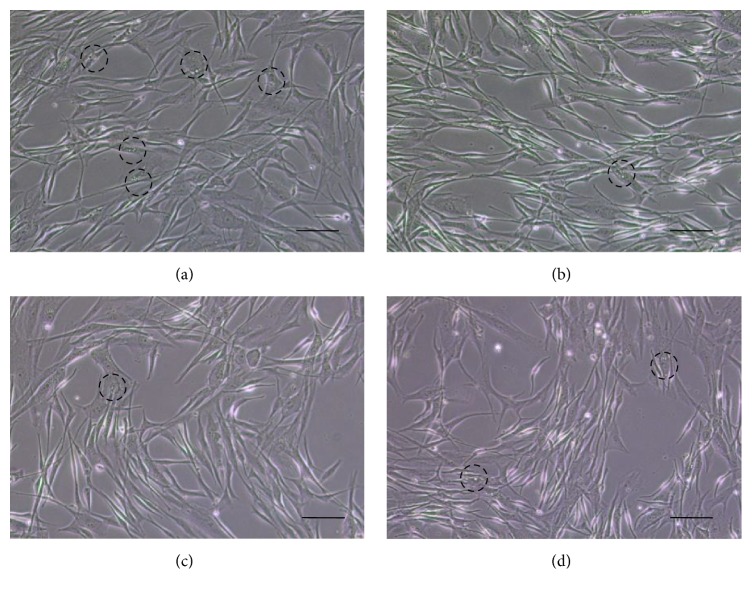
Representative images of melanocytes after 3 days cultured in differentiation media. (a) Control, (b) 10 nMSH, (c) 2.4 *μ*g/mL OSA, and (d) 30 *μ*g/mL RBE. Original magnification: (a)–(d) ×100; scale bar: 100 *μ*m.

**Figure 2:**
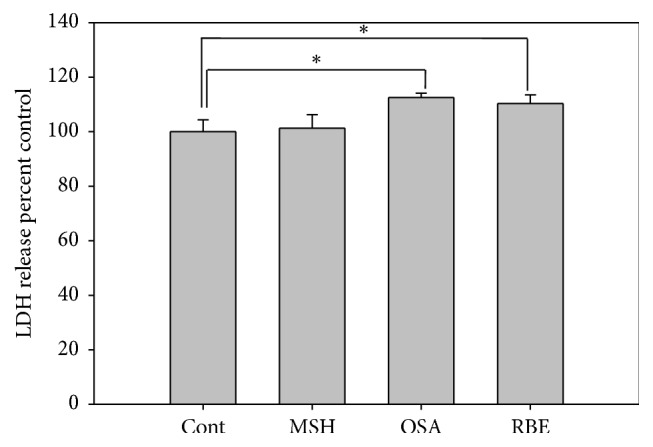
Representative graph of the LDH assay in melanocytes after 3 days cultured in differentiation media and after 3 days cultured in MSH, OSA, and RBE enriched media. Significant differences were determined by Student's* t*-test; ^*∗*^ *P* < 0.05.

Compared to the cells in the MSH, OSA, and RBE, those in the control group showed a greater number of vacuoles in the cytoplasm, indicating that these cells had aged more (Figure 1(a), dot circle). Furthermore, the MSH, OSA, and RBE cells showed more bipolar dendritic processes than the control cells and no apoptotic or necrotic cell death (Figure 1). However, OSA and RBE cells had 10% greater LDH activity, compared to the control group (Figure 2), indicating cell stress.

### 3.2. Melanin Contents and Tyrosinase Activity

**Figure 3:**
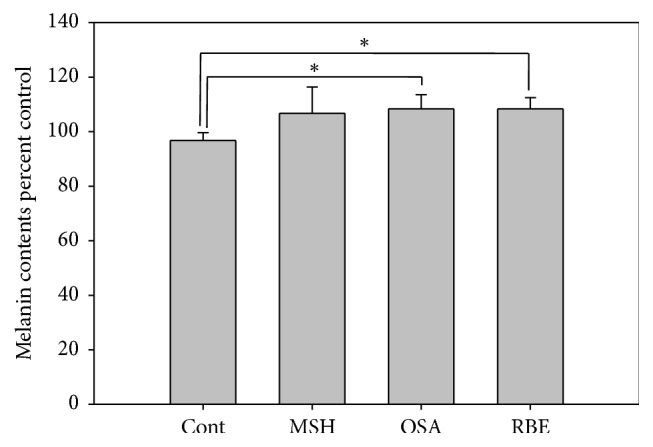
Representative graph of melanin contents in melanocytes after 3 days cultured in differentiation media and after 3 days cultured in MSH, OSA, and RBE enriched media. Significant differences were determined by Student's* t*-test; ^*∗*^ *P* < 0.05.

**Figure 4:**
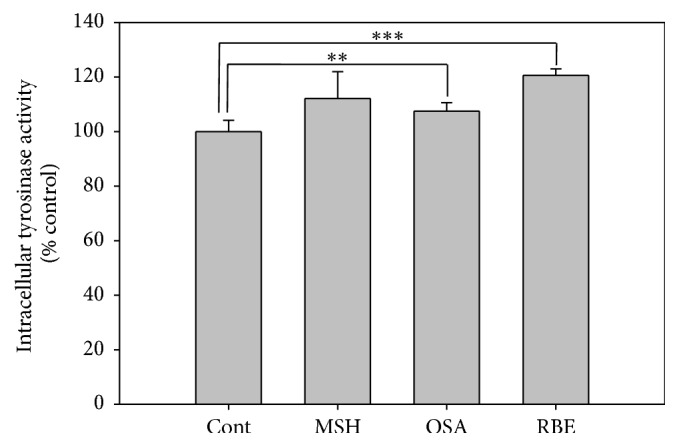
Representative graph of tyrosinase activity in melanocytes after 3 days cultured in differentiation media and after 3 days cultured in MSH, OSA, and RBE enriched media. Significant differences were determined by Student's* t*-test; ^*∗∗*^ *P* < 0.01; ^*∗∗∗*^ *P* < 0.005.

Melanin levels were 10% higher in the MSH, OSA, and RBE groups compared to the control group (Figure 3). Also, tyrosinase activity was approximately 10% higher in the OSA and MSH groups and 20% higher in the RBE group compared to the control group (Figure 4).

### 3.3. Protein Expression Levels

**Figure 5:**
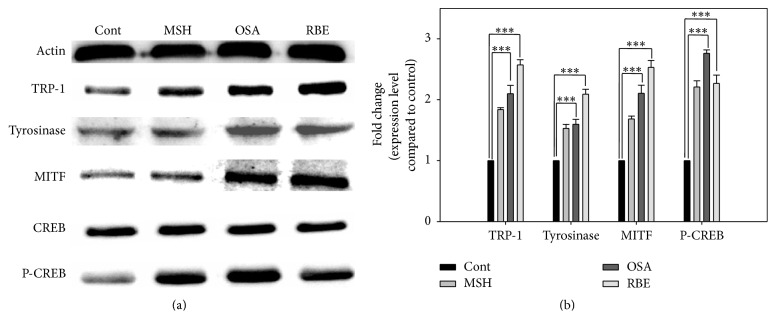
Melanogenesis effect in melanocytes after OSA and RBE were added and the cells cultured for 3 days. TRP-1, tyrosinase, MITF, and P-CREB increased remarkably compared to the control group. Significant differences were determined by Student's* t*-test; ^*∗∗∗*^ *P* < 0.005.

Protein expression of the melanogenesis markers TRP-1, tyrosinase, and MITF was assessed by Western blotting. As shown in Figure 5, TRP-1 and MITF increased 2.5-fold in the RBE group and 2-fold in the OSA group compared to the control. Tyrosinase increased 2-fold and 1.5-fold in the RBE and OSA group, respectively, compared to the control. This result suggested that OSA and MSH had comparable efficacy to stimulate melanogenesis, whilst RBE had greater efficacy than MSH. Furthermore, the P-CREB level increased approximately 2-fold in the MSH, OSA, and RBE groups, suggesting that melanin synthesis occurred via CREB phosphorylation.

### 3.4. Histological Evaluation and Immunohistochemistry

**Figure 6:**
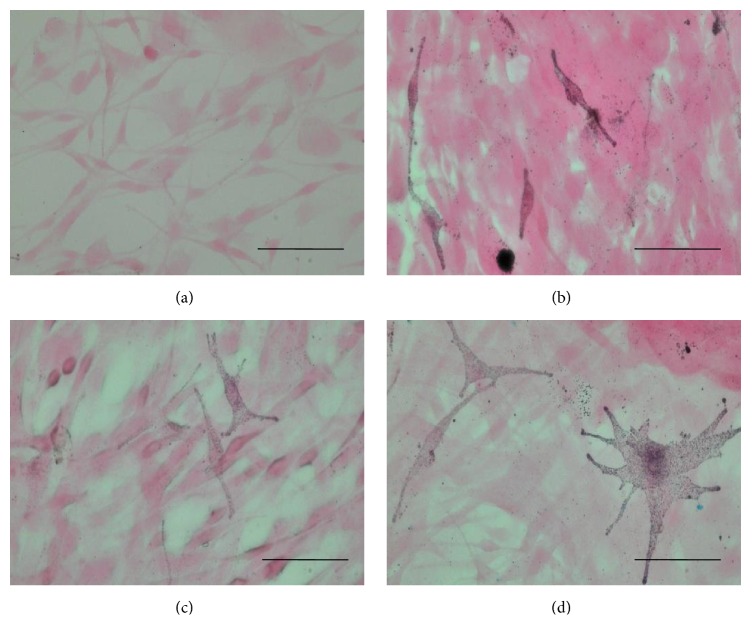
Representative images of Fontana-Masson stained melanocytes after 3 days cultured in differentiation media (dark color indicates secreted melanin). (a) Control, (b) 10 nMSH, (c) 2.4 *μ*g/mL OSA, and (d) 30 *μ*g/mL RBE. Original magnification: (a)–(d) ×400; scale bar: 50 *μ*m.

**Figure 7:**
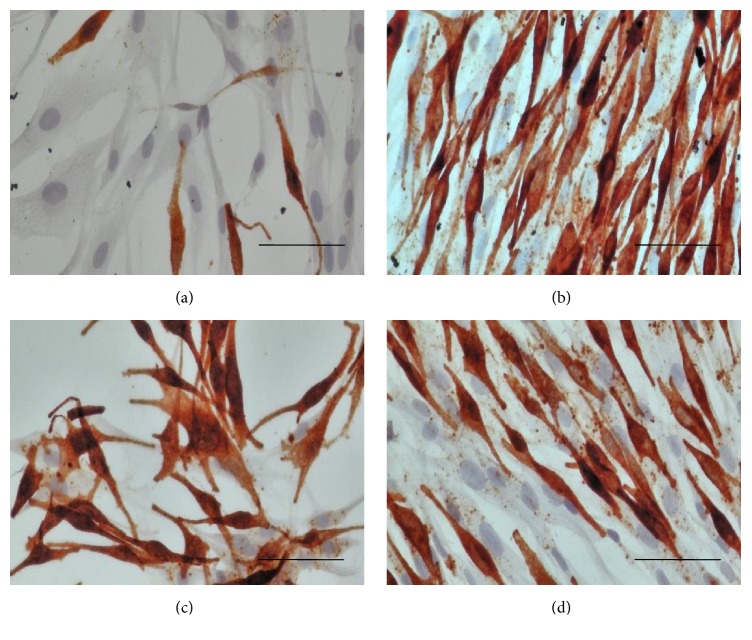
Representative images of HMB-45 immunostained melanocytes after 3 days cultured in differentiation media (brown color indicates melanosome). (a) Control, (b) 10 nMSH, (c) 2.4 *μ*g/mL OSA, and (d) 30 *μ*g/mL RBE. Original magnification: (a)–(d) ×400; scale bar: 50 *μ*m.

Fontana-Masson staining is used to confirm the fact that melanocytes are synthesizing melanin [15]. In this study, MSH, OSA, and RBE groups were positive for Fontana-Masson staining, whilst the control group was negative (Figure 6). Similarly, MSH, OSA, and RBE groups were positive for HMB45, whilst the control group was negative (Figure 7) (Table 2). HMB45 is targeted to the melanosome [16]. Localized HMB45 staining indicates active melanosome formation and, thus, melanocyte differentiation, suggesting that HMB45 is a melanocytic activation marker [14].

## 4. Discussion

Several researchers have used RBE to activate various types of cells [17–19]. Hagl et al. found that RBE, which contains oryzanols and tocopherol components, compensated age-related mitochondrial dysfunction by increasing the mitochondrial respiration and membrane potential in a neurodegenerative mouse model. Choi et al. showed that the linoleic acid and *γ*-oryzanol components in RBE promoted hair growth by inducing hair follicles into the anagen stage (active growth phase) and increasing VEGF, IGF-1, and KGF expressions. Also, Fukumoto et al. reported that RBE had the ability to differentiate mesenchymal stem cells into osteogenic cells. These previous studies [17–19] were mainly focused on the organic compounds in rice bran extracts, such as vitamins, oryzanols, tocopherols, tocotrienols, and linoleic acid.

However, besides its organic compounds, rice bran is rich in minerals, such as Si, K, and Mg. Liu et al. reported that rice bran ash contains various mineral components, Si, C, K_2_O, CaO, Na_2_O, MgO, Al_2_O_3_, ZnO, MnO_2_, and Fe_2_O_3_, with Si accounting for 65 wt% [20]. Hence, this study focused on RBE minerals, in particular silicic acid. The composition of RBE (Table 1) was similar to that of the rice bran ash studied by Liu et al. [20], suggesting that 30 *μ*g/mL of RBE contained 5 *μ*g/mL of soluble Si.

Silicate usually exists as SiO_2_, which has shown cytotoxicity in human cell lines (A431 and A549) [4]. Furthermore, in a rat liver cell line, SiO_2_ decreased cell viability in a dose-dependent manner [5]. In this study, there were no signs of apoptosis or cell damage on melanocytes (Figure 1) at 0.24 *μ*g/mL OSA and 30 *μ*g/mL RBE. Melanocytes were apparently normal in terms of morphology. It was, however, shown that they were stressed a little in the test of LDH activity.

Several studies have investigated the effects of OSA, which is less cytotoxic than silicate. For example, Dong et al. found that OSA significantly increased type 1 collagen and osteocalcin in the osteoblast-like cell lines MG-63 and U2-OS [7]. Similarly, Reffitt et al. showed increased type 1 collagen, alkaline phosphatase, and osteocalcin synthesis in osteoblast-like cell lines, MG-63 and HCCl [6]. Also, Grotheer et al. showed that OSA-releasing hydrogel decreased the inflammatory response and aided chronic wound recovery by inhibiting NF-*κ*B expression or enhancing I*κ*B, NF-*κ*B-inhibiting protein [11].

In this study, we investigated the effect of OSA and RBE mineral components on melanocytes. OSA and RBE increased tyrosinase activity and melanin content, and both materials activated TRP-1, tyrosinase, MITF, and P-CREB in melanocytes.

Melanin synthesis increased in MSH, OSA, and RBE groups compared to the control group. Hence, melanin contents were elevated in MSH, OSA, and RBE groups (Figure 3) and Fontana-Masson staining confirmed that melanin was present in the melanocytes (Figure 6). These results suggest that OSA and RBE were able to stimulate the melanocytes to synthesize melanin. Moreover, OSA and RBE groups had 10 and 20% higher tyrosinase activity than control group. Tyrosinase catalyzes melanin synthesis by oxidizing tyrosine to DOPA and the dehydrogenation of DOPA to dopaquinone [21]. In this study, RBE was more efficient at catalyzing melanin synthesis than MSH.

The Western blotting results showed that TRP-1, tyrosinase, and MITF expression levels were higher in the OSA and RBE groups compared to the control group (Figure 5). TRP-1 oxidizes 5,6-dihydroxyindole-2-carboxylic acid (DHICA) to a carboxylated indole-quinone [22], which is one of the processes in melanin synthesis. Furthermore, MITF induces upregulation of the tyrosinase gene family [23]. The increase in MITF is associated with the HMB45 immunostaining results. This is because PMEL is involved in the deposition of melanin onto the internal fibrils of the melanosome, HMB45 staining is localized in melanosomes, and PMEL expression is transcriptionally regulated by MITF [16]. Hence, HMB45 can be used as a melanogenesis marker in melanocytes [16]. Thus, the positive HMB45 staining in OSA and RBE groups is due to the increased level of MITF. In addition, increased MITF expression level can be explained by the increased P-CREB level. Bu et al. showed that inhibition of CREB phosphorylation consequently decreased MITF expression in a B16-F10 melanoma cell line [24].

## 5. Conclusion

Melanogenesis is based on a complicated mechanism regulated by multiple factors. Among them, TRP-1, tyrosinase, and MITF are regarded as key melanogenic factors. In this study, we showed that OSA has the potential to promote melanogenesis and both RBE and OSA have similar cell viability, protein expression, and immunostaining results, suggesting that RBE comprises specific minerals which promote melanin synthesis through MITF and CREB phosphorylation. Therefore, RBE could be used as a novel material to promote melanin synthesis by stimulating melanocytes with its soluble Si and mineral components.

## Figures and Tables

**Table 1:** Chemical composition of RBE.

Element	Si	Ca	Mg	K	Na	P
mg/kg (solution)	180	5	17	2930	113	300

**Table 2:** Results of staining after OSA and RBE treatment on melanocyte.

	Cont	MSH	OSA	RBE
FM stain	−	++	+	+
HMB45	+	++++	+++	+++
